# Characteristics, Outcomes and Mortality Risk Factors of Pediatric In-Hospital Cardiac Arrest in Western China: A Retrospective Study Using Utstein Style

**DOI:** 10.3390/children12050579

**Published:** 2025-04-29

**Authors:** Jiaoyang Cao, Jing Song, Baoju Shan, Changxin Zhu, Liping Tan

**Affiliations:** 1 Department of Emergency, Children’s Hospital of Chongqing Medical University, National Clinical Research Center for Child Health and Disorders, Ministry of Education Key Laboratory of Child Development and Disorders, Chongqing Key Laboratory of Child Rare Diseases in Infection and Immunity, Chongqing 401123, China; caojiaoyang@hospital.cqmu.edu.cn (J.C.); shanbaoju@hospital.cqmu.edu.cn (B.S.); 2 College of Pediatrics, Chongqing Medical University, Chongqing 400016, China; songjing1@stu.cqmu.edu.cn (J.S.); zhuchangxin@stu.cqmu.edu.cn (C.Z.)

**Keywords:** pediatric, in-hospital cardiac arrest, Utstein style, mortality risk factors, CPR outcomes

## Abstract

**Background**: Pediatric in-hospital cardiac arrest (IHCA) remains a critical health challenge with high mortality rates. Limited data from Western China prompted this study to investigate the characteristics of IHCA using the Utstein style. **Methods**: A retrospective analysis of 456 pediatric patients with IHCA (2018–2022) at the Children’s Hospital of Chongqing Medical University assessed demographics, arrest characteristics, outcomes and mortality risk factors. The primary outcome was survival to discharge; the secondary outcomes included return of spontaneous circulation (ROSC) > 20 min, 24 h survival, and favorable neurological outcomes. Logistic regression was used to identify the mortality risk factors. **Results:** ROSC > 20 min was achieved in 78.07% of cases, with 37.94% surviving to discharge (86.13% of survivors had favorable neurological outcomes). Etiological stratification identified general medical conditions (52.63%) as the predominant diagnoses, with surgical cardiac patients demonstrating superior resuscitation outcomes (ROSC > 20 min: 86.84%, discharge survival: 64.04%). Initial arrest rhythms predominantly featured non-shockable patterns, specifically bradycardia with poor perfusion (79.39%), whereas shockable rhythms (ventricular fibrillation/pulseless ventricular tachycardia) constituted only 4.17% of cases. Multivariable regression analysis identified five independent risk factors: vasoactive infusion before arrest (OR = 7.69), CPR > 35 min (OR = 13.92), emergency intubation (OR = 5.17), administration of >2 epinephrine doses (OR = 3.12), and rearrest (OR = 8.48). Notably, prolonged CPR (>35 min) correlated with higher mortality (8.96% survival vs. 48.54% for 1–15 min), yet all six survivors with CPR > 35 min had favorable neurological outcomes. **Conclusions**: These findings underscore the persistent challenges in pediatric IHCA management while challenging the conventional CPR duration thresholds for futility. The identified mortality risk factors inform resuscitation decision making and future studies.

## 1. Introduction

Pediatric in-hospital cardiac arrest (IHCA) represents a significant global health challenge and is often associated with substantial mortality. The incidence of IHCA varies considerably owing to regional disparities, such as those between developed and developing countries as well as between rural and urban areas, with differences in the documentation of cardiac arrest (CA) occurrences and outcomes. In the United States, approximately 15,200 children are estimated to undergo cardiopulmonary resuscitation (CPR) for IHCA annually [1]. Previous studies have reported that CA occurs in 0.18–3% of pediatric hospital admissions and 1.8–6% of pediatric intensive care unit admissions [2,3,4,5,6], representing significant societal, familial, and economic costs.

Owing to advancements in early recognition, high-quality CPR, post-arrest care, and the implementation of extracorporeal cardiopulmonary resuscitation (ECPR), pediatric outcomes have improved. A multicenter observational study of 7433 hospitalized children who received CPR between 2000 and 2018 showed that survival rates increased from 19% to 38%, with a peak of 40% in 2011 [7]. Furthermore, the American Heart Association (AHA) recently reported that survival to discharge after pulseless IHCA in children increased from 18.9% to 44.2% between 2000 and 2022 in the Get With The Guidelines (GWTG) database [8]. However, survival rates have plateaued since 2010, and more than half of children with IHCA do not survive to hospital discharge [4,7,8,9,10,11,12].

Pediatric IHCA is a modifiable disease process influenced by factors, such as patient diagnosis, immediate cause of the event [13], initial rhythm [13,14], CPR quality and hemodynamic achievement [12], CPR duration [2,15], and post-arrest care [16]. Understanding the characteristics of IHCA can aid in early identification and intervention, thereby improving CPR success rates and prognostic evaluations. Compared to decades of continuous monitoring and improvement in Europe and the United States, China’s exploration of the national epidemiology and treatment quality of cardiac arrest has just begun. The BASIC registry, China’s first nationwide cardiac arrest study, offers valuable insights and facilitates international comparisons of cardiac arrest strategies. However, data on pediatric IHCA in Western China are lacking [17,18,19].

We conducted a retrospective study using the Utstein report style at a large tertiary academic children’s hospital in Chongqing to examine the characteristics and mortality risk factors of pediatric IHCA in Western China. This study aimed to enhance CPR outcomes and provide preliminary data for future large-scale multicenter resuscitation resI confirmearch.

## 2. Materials and Methods

### 2.1. Study Design

This single-center, retrospective, observational study was performed at the Children’s Hospital of Chongqing Medical University in China, a National Child Health and Disease Clinical Research Center with a capacity of 2480 beds. Pediatric patients who experienced IHCA and received CPR after admission between 1 January 2018 and 31 December 2022 were included in this study. This study was approved by the Institutional Review Board of the Children’s Hospital of Chongqing Medical University, and the requirement for written informed consent was waived.

### 2.2. Data Collection and Outcomes

Data were collected from medical records and Utstein style CPR reports completed by trained medical professionals. This study used variables based on the in-hospital Utstein style, categorized into three groups: patient, pre-event, and CA process [20]. The patient variables included age, sex, and locality. The pre-event variables were prehospital arrest, illness category, time of hospital admission, and interventions already in place (e.g., vasoactive infusion, tracheal tube, and mechanical ventilation). The CA process variables were the time of events (beginning and end of CPR), arrest location, procedures performed (e.g., chest compression, airway interventions, and defibrillation), initial rhythm (bradycardia with poor perfusion, ventricular fibrillation, pulseless ventricular tachycardia, asystole, and pulseless electrical activity), medications used (epinephrine, sodium bicarbonate, atropine, calcium, creatine phosphate sodium, and vitamin C), reasons for stopping CPR, pediatric cerebral performance category (PCPC) scores at admission and discharge (category 1: normal age-appropriate neurodevelopmental functioning; category 2: mild cerebral disability; category 3: moderate cerebral disability; category 4: severe disability; category 5: coma/vegetative state; and category 6: brain death) [21], and time of death.

Patients were categorized into five illness groups according to the Utstein guidelines [20]: general medical, including all non-heart-related medical conditions; general surgery, including all noncardiac-related and nontraumatic surgical procedures; surgical cardiac, including all open-heart surgeries, with or without cardiopulmonary bypass, to treat coronary heart diseases, valve, aortic, and congenital diseases; medical cardiac, including all nonsurgical cardiac conditions managed medically or via noninvasive interventions; and trauma, including all conditions caused by external forces that required urgent evaluation or surgical intervention.

The primary outcome was survival to hospital discharge. The secondary outcomes included return of spontaneous circulation (ROSC) > 20 min, 24 h survival, and survival to discharge with favorable neurological outcomes. Neurological outcomes were determined using the PCPC scale [20]. A favorable neurological outcome was defined as a PCPC category of 1, 2, or 3 at hospital discharge or a discharge PCPC no worse than that on admission.

### 2.3. Inclusion and Exclusion Criteria

All admitted patients aged 29 days to 18 years who sustained a clinical event that required CPR for at least 1 min were included. We excluded patients who experienced IHCA following requests from legal guardians to discontinue all treatment measures and those with incomplete medical records. Consistent with the Utstein style registry guidelines, only the first in-hospital index of cardiac arrest and resuscitation was described and analyzed for patients with multiple cardiac arrests.

### 2.4. Statistical Analysis

Data analysis was performed using SPSS software (version 30.0). Categorical variables are presented as frequencies and percentages, and continuous variables are presented as medians with interquartile ranges (25th and 75th percentiles). Continuous variables, such as age, duration of CPR, time from hospital admission to event, and number of epinephrine doses, were converted into categorical variables based on prior studies or median values [2,5,12,15]. Comparative analyses of categorical data were performed using the chi-square test or chi-square test for trends. Univariate and multivariable logistic regression analyses were conducted to identify the factors associated with mortality risk. Variables with *p* ≤ 0.1 in the univariate analysis were entered into the multivariable model, with *p* ≤ 0.05 considered indicative of statistical significance. Dummy variables were used for multiple categorical variables. If any dummy variable demonstrated statistical significance in relation to the dependent variable, all dummy variables within the corresponding group were incorporated into the multivariable regression model. Odds ratios (ORs) are presented with 95% confidence intervals (CIs).

## 3. Results

**Figure 1:**
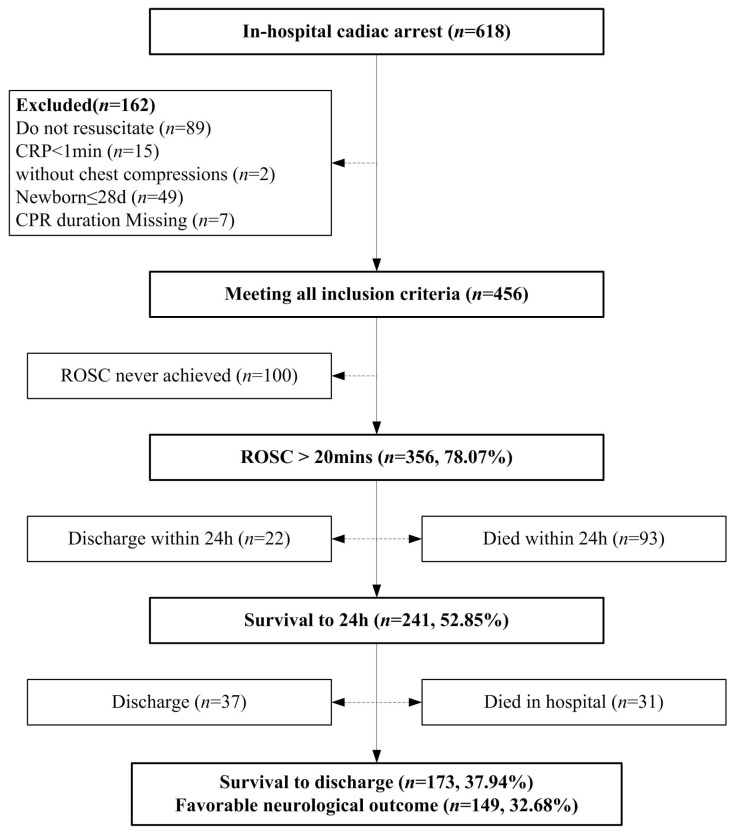
Utstein style flow diagram of patients evaluated, CPR events, and overall outcomes.

A total of 618 patients underwent IHCA at the Children’s Hospital of Chongqing Medical University between 1 January 2018 and 31 December 2022. As shown in Figure 1, 162 cases were excluded because they did not undergo CPR (*n* = 89): pre-arrest do-not-resuscitate (DNR) directives or family refusal of resuscitation (*n* = 21), withdrawal of life-sustaining therapy (WLST) prior to arrest *(n* = 67), or spontaneous return of circulation without intervention (*n* = 1); CPR duration < 1 min (*n* = 15); without chest compressions (*n* = 2); age ≤ 28 days (*n* = 49); and missing CPR duration (*n* = 7). Thus, 456 patients were included in the final analysis. Of the 456 patients, 356 (78.07%) attained ROSC > 20 min, 241 (52.85%) were still alive 24 h after the event, 173 (37.94%) survived to hospital discharge, and 149 (32.68%) had favorable neurological outcomes (86.13% of hospital survivors). Additionally, 59 (12.93%) patients were discharged after withdrawal owing to end-stage diseases, poor prognosis, or economic issues.

**Figure 2:**
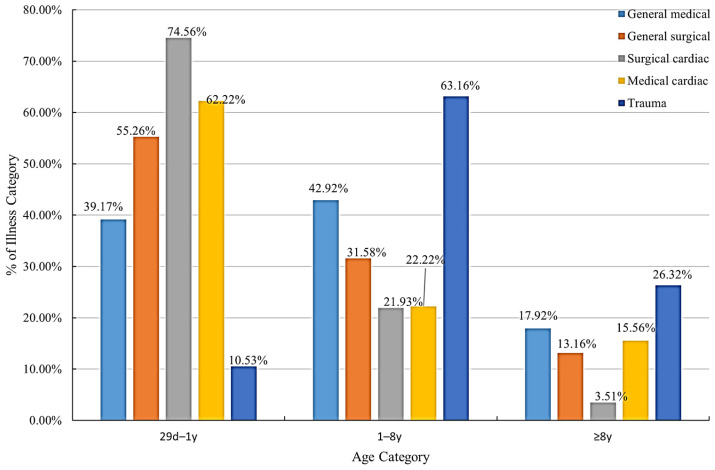
Illness distribution by age category.

Patient demographics and arrest characteristics are presented in Table 1. The median age was 0.98 (0.33, 4.31), with 253 males and 203 females. Of the patients, 50.44% were <1 year of age. There were 34.87% cardiac, 60.96% noncardiac, and 4.17% trauma cases. Patients who underwent cardiac surgery were most prevalent in the infant group (Figure 2). Arrests were most frequent in the pediatric intensive care unit (54.39%), followed by the inpatient ward (30.48%) and cardiac surgery intensive care unit (9.43%). A total of 32.89% of arrests occurred after 24 h of hospitalization, and 39.47% occurred after 1 week. The median CPR duration was 8 (3,25) minutes and ranged from 1 to 220 min, with most patients receiving CPR for 1–15 min (67.76%) and 14.69% receiving more than 35 min. Among the initial rhythms, bradycardia with poor perfusion was the most common (79.39%), whereas only 4.17% of patients presented with an initial shockable rhythm. Defibrillation was performed in 7.24% of patients. Regarding interventions, 57.46% of the patients were intubated before cardiac arrest, 28.07% underwent emergency intubation, and 14.47% received only bag-mask ventilation (BMV) during CPR. Epinephrine was the most frequently administered medication during CPR (81.14%), with a median dose of 2 (1, 5). In this study, 136 (29.82%) children experienced multiple CPR events during hospitalization, 83/136 had only one additional CPR event, and 53/136 had more than one event.

**Figure 3:**
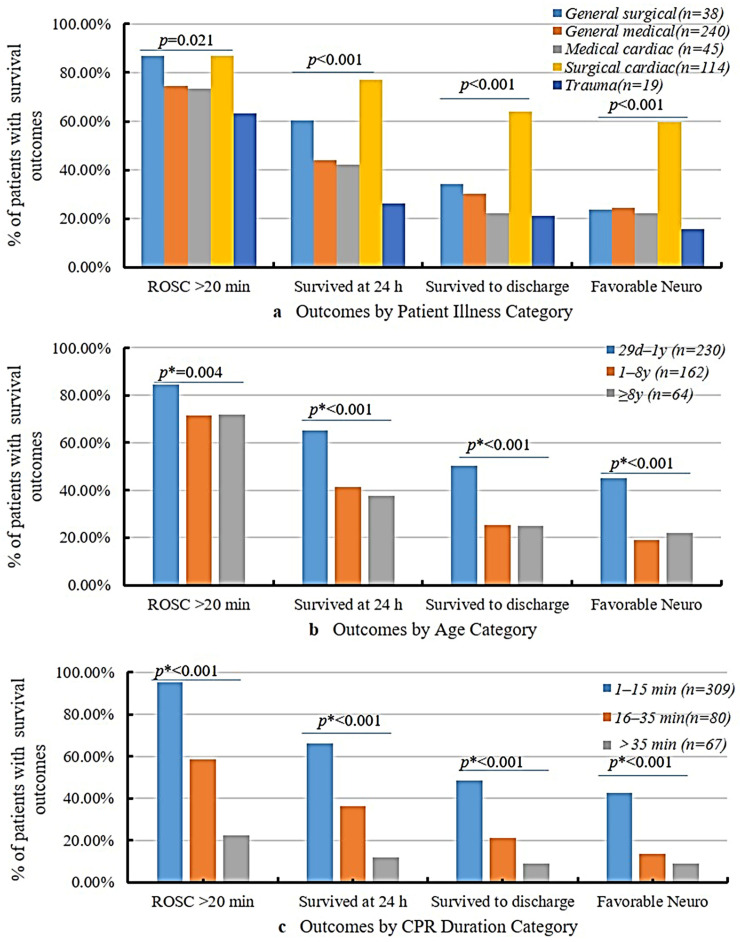
Outcomes by patient illness, age, and CPR duration category. *p* value for differences in outcome relative to the categorized patient illness from chi-square test; *p* * values from Mantel–Haenszel chi-square test.

Illness, age, and CPR duration influenced the survival rates and neurological outcomes (Figure 3). Patients who underwent cardiac surgery showed the best outcomes with ROSC > 20 min (86.84%), and their favorable neurological outcome was 59.65%, which was much higher than that of patients who experienced trauma (15.79%). The infant group (29 d–1 y) demonstrated better outcomes than the other two age groups. Moreover, CPR duration is a key determinant of prognosis. Patients with CPR durations of 1–15 min had ROSC > 20 min at 95.15% and favorable neurological outcome at 42.72%; however, only six children (8.96%) with a CPR duration > 35 min survived to discharge, and all six survivors exhibited favorable neurological outcomes.

Multivariable logistic regression was conducted on factors with *p* ≤ 0.1 from the univariate analysis to identify mortality risk factors, with independently associated factors shown in Table 2. Vasoactive infusion before arrest (OR = 7.69, *p* < 0.001), CPR duration > 35 min (OR = 13.92, *p* < 0.001), emergency intubation at arrest (OR = 5.17, *p* = 0.008), number of epinephrine doses > 2 (OR = 3.12, *p* = 0.026), and rearrest (OR = 8.48, *p* < 0.001) were independent risk factors for mortality. Patients were more likely to survive if their disease classification was surgical cardiac (OR = 0.06, *p* < 0.001) or if the first rhythm was VF/pVT (OR = 0.15, *p* = 0.010).

## 4. Discussion

This retrospective observational study, conducted at a tertiary pediatric hospital in Chongqing, Western China, analyzed 456 pediatric IHCA cases between 2018 and 2022. This study comprehensively examined IHCA characteristics, treatment modalities, and mortality risk factors, addressing the paucity of data in this region. These findings underscore the necessity of region-specific insights to optimize resuscitation strategies and enhance patient outcomes.

In this study, 78.07% of patients attained ROSC > 20 min, a rate comparable to or exceeding international benchmarks [2,4,10,12,22,23,24]. The survival-to-discharge rate was 37.94%, higher than that in previous reports from China [4] but lower than that in developed countries [2,7,8,9,10,11,12,20,22,23,24,25,26]. Among survivors, 86.13% exhibited favorable neurological outcomes, as assessed by the PCPC, aligning with international studies reporting favorable outcomes in approximately 90% of cases [2,12].

The median age of 0.98 years and the high prevalence of IHCA in infants (50.44%) align with global trends, highlighting the heightened vulnerability of this age group [25,27]. Consistent with prior studies, survival-to-discharge rates declined with increasing age [16,26,28]. Infants demonstrated the highest survival rate (47.5%) compared to adolescents (26.89%) [26]. Schleien et al. reported significantly higher mortality in children aged > 1 year (68%) versus infants (44%) [28]. Similarly, our study found that infants had superior survival and neurological outcomes compared with children aged 1–8 and ≥8 years. However, age was not an independent mortality risk factor in the multivariate analysis, likely due to age-specific disease distributions, such as the higher prevalence of surgical cardiac conditions with better prognoses in infants than in older children.

In our study, general medical conditions were the leading etiology of the IHCA. Surgical cardiac patients exhibited the highest event survival and discharge survival rates, with surgical cardiac disease identified as an independent protective factor against mortality, consistent with prior studies [3,29]. The reported discharge survival rates after cardiac arrest in patients undergoing heart operations range from 37% to 69% [15,30,31,32,33,34]. One study noted improved survival across all illness categories over 20 years, with the greatest increase among surgical cardiac patients [29], attributed to integrated preoperative and postoperative management, the application of ECPR technology, easier intervention of arrhythmias caused by structural heart disease, and advanced ICU monitoring and care [29,35].

Our study on IHCA showed different causes than those of out-of-hospital cardiac arrest (OHCA). In children, OHCA is often due to preventable trauma (such as traffic accidents, violent events, falls from heights, and drowning) [36]. In contrast, our IHCA cases had more general medical diseases (e.g., sepsis, respiratory failure) and perioperative complications. For example, 25.00% of our IHCA cases were related to cardiac surgery, whereas only 4.17% were due to trauma. This highlights the need for tailored prevention strategies; IHCA focuses on early detection in high-risk hospitalized patients, while OHCA focuses on community injury prevention.

Bradycardia with poor perfusion was the most common initial rhythm (79.39%) in our study, followed by asystole/PEA (10.31%), while VF/pVT accounted for only 4.17% of the cases. Consistent with prior research, only ~10% of pediatric cardiac arrests present with an initial shockable rhythm [9]. Non-shockable rhythms, particularly bradycardia with poor perfusion, dominate in over 50% of IHCA cases [9,27,37]. The high prevalence of bradycardia and low prevalence of shockable rhythm align with the asphyxial nature of pediatric IHCA, where arrest culminates from progressive hypoxemia or shock rather than primary arrhythmias [14,38]. This underscores the imperative for early recognition of pre-arrest compensatory phases to guide timely interventions. Our findings corroborate earlier studies showing that pediatric patients with initial VF or pVT rhythms have a lower mortality risk [13,14,39], likely due to the greater efficacy of defibrillation in restoring effective cardiac rhythm.

Previous studies have established that CPR duration is independently associated with patient outcomes. Matos et al. reported that for CPR durations of 1–15 min, survival rates decreased linearly by 2.1% per minute, favorable neurological outcomes declined by 1.2% per minute, and discharge survival was approximately 41%. For CPR >35 min, survival dropped to 12% [15]. A systematic review and meta-analysis further confirmed a steady decline in favorable neurological outcomes with each 5 min increase in CPR duration, with significant reductions observed beyond 11–15 min [40]. Similarly, our study found that prolonged CPR duration significantly reduced discharge survival rates: 48.54% for CPR < 15 min, 21.25% for 16–35 min, and 8.96% for >35 min. CPR time > 35 min was identified as an independent risk factor for pediatric IHCA mortality. Although survival rates are lower with extended CPR, it is not entirely futile, as some patients achieve favorable neurological outcomes [15]. In our cohort, six patients with CPR > 35 min survived to discharge, all with favorable neurological outcomes. In China, physicians may recommend stopping resuscitation after 30 min without ROSC, but the final decision rests with the family [17]. These findings highlight the ongoing uncertainty regarding the optimal CPR duration.

Tracheal intubation is the standard of care during IHCA and is often prioritized because of the high prevalence of underlying pulmonary or airway diseases in pediatric IHCA, suggesting a respiratory etiology [22,41]. However, intubation during CPR carries the risk of complications and adverse events. In our study, emergency intubation was an independent mortality risk factor compared to bag-mask ventilation (BMV). A large retrospective study of pediatric patients with IHCA without advanced airways found lower survival rates in intubated versus non-intubated cases (36% vs. 41%, RR 0.89, *p* = 0.03) [42]. Similar findings have been reported in pediatric OHCA, where endotracheal intubation yielded worse outcomes than BMV [43]. This may stem from CPR interruptions during intubation [44]; delays in defibrillation, vascular access, or epinephrine administration [45,46]; as well as hyperventilation and increased intrathoracic pressure, which are associated with poorer outcomes in animal models [47,48]. Additionally, unrecognized esophageal intubation can compromise oxygenation and ventilation. The optimal airway management strategy during cardiac arrest, particularly in pediatric IHCA, remains uncertain.

Epinephrine has been used for cardiorespiratory arrest since the 1960s [49]. It enhances coronary perfusion pressure, improving the likelihood of ROSC. However, repeated doses increase beta-adrenergic activity and oxygen demand, potentially impairing myocardial function and precipitating ventricular tachycardia or fibrillation [50]. The current AHA guidelines recommend administering epinephrine at 0.01 mg/kg (maximum 1 mg) as soon as possible, with subsequent doses every 3–5 min [51]. In this study, epinephrine was administered according to the AHA guidelines, with 38.16% of patients (*n* = 174) receiving more than two doses. Repeated epinephrine administration (>2 doses) was identified as an independent mortality risk factor, consistent with studies showing higher mortality in patients requiring frequent adrenaline due to severe illness [22,35,52,53].

Rearrest was also identified as an independent risk factor for mortality in the present study. It is a critical determinant of outcomes and frequently occurs in patients who recover from spontaneous circulation after initial resuscitation [54]. The incidence and associated risk factors for rearrest remain poorly characterized. Youping Zhang et al. discovered that rearrest is a significant independent risk factor for death in IHCA patients, and the incidence of rearrest was high up to 61.8%; the OR for death in rearrest patients was 11.493 (95% CI 5.603 to 25.309) compared with single arrest patients [55].

At our institution, all clinical staff members must complete Basic Life Support (BLS) training. Resuscitation team members are also required to obtain biannual Pediatric Advanced Life Support (PALS) certification and participate in quarterly IHCA simulations focusing on early bradycardia and hypoxia reversal. Our retrospective study design did not allow for a direct analysis of the impact of training on outcomes, but we recognize the importance of standardized pediatric emergency training in improving IHCA management. However, competency metrics were not systematically documented in this study; therefore, we could not quantify the association between training and survival rates. Future prospective studies should integrate such data, as recommended by the European Resuscitation Council’s 2021 guidelines [56], which emphasize ongoing education for healthcare providers. This will help separate the training effects from patient risk factors and refine resuscitation protocols.

The present study had some limitations. First, it was limited by its single-center, retrospective design and small sample size. We documented only the classification of underlying diseases but did not collect granular severity-grading information. While the time to CPR initiation is a key factor for outcomes, our data sources exhibited significant missing data in time-stamped documentation (approximately 91.67% of cases lacked specific time annotations, and the time point was replaced by “immediately” in the rescue record). Consequently, this variable could not be included in the multivariable analysis. Future prospective studies should prioritize the incorporation of disease severity grading and time-sensitive indicators. Second, we only analyzed the characteristics of the initial IHCA and lacked information on post-cardiac arrest syndrome (PCAS). Early post-arrest interventions, such as targeted temperature management (TTM), were not evaluated in this study and may have affected outcomes. Third, the only marker of neurological outcome was the PCPC score. This is a limited tool that is typically extrapolated retrospectively from medical record reviews and lacks sensitivity for more granular neurological dysfunctions [27]. Finally, the outcomes for patients who were discharged against treatment were not established, and these patients were excluded from multivariable logistic regression analysis. This led to a small number of patients in the analysis of mortality risk factors.

## 5. Conclusions

This is the first Utstein style report of pediatric IHCA in Western China. A significant proportion of pediatric patients with IHCA did not survive to discharge, highlighting the need for improved resuscitation strategies to enhance outcomes. Among the survivors, neurological outcomes were favorable in the majority of patients, and all six survivors who received CPR for >35 min exhibited favorable neurological outcomes. Performing CPR for >35 min is not futile in some patients. Risk and protective factors for mortality have been identified and may be used to guide decision making during resuscitation.

## Figures and Tables

**Table 1:** Patient demographics and arrest characteristics.

Variables	Overall *n* = 456	Variables	Overall *n* = 456
Age, y, Median (quartiles)	0.98 (0.33, 4.31)	Weekend ^1^, *n* (%)	144 (31.58)
Age category, *n* (%)		Night ^2^, *n* (%)	200 (43.86)
29 d–1 y	230 (50.44)	First documented pulseless rhythm, *n* (%)	
1–8 y	162 (35.53)	Bradycardia with poor perfusion	362 (79.39)
≥8 y	64 (14.04)	Asystole/PEA	47 (10.31)
Male, *n* (%)	253 (55.48)	VF/pVT	19 (4.17)
Local, *n* (%)	235 (51.54)	Other or Unknown	28 (6.14)
Illness Category, *n* (%)		Duration of CPR, min, Median (quartiles)	8 (3, 25)
General medical	240 (52.63)	Category of CPR duration, min, *n* (%)	
General surgical	38 (8.33)	1–15	309 (67.76)
Surgical cardiac	114 (25.00)	16–35	80 (17.54)
Medical cardiac	45 (9.87)	>35	67 (14.69)
Trauma	19 (4.17)	Airway management, *n* (%)	
Prehospital arrest, *n* (%)	51 (11.18)	BMV	66 (14.47)
Interventions in place before arrest		ETI in place before arrest	261 (57.24)
Respiratory support, *n* (%)		Emergency intubation at arrest	129 (28.29)
Invasive mechanical ventilation	264 (57.89)	Pharmacological interventions	
Noninvasive ventilation	16 (3.51)	Epinephrine, *n* (%)	370 (81.14)
None	176 (38.60)	Number of epinephrine doses, median	2 (1, 5)
Vasoactive infusion, *n* (%)	223 (48.90)	Number of epinephrine doses, *n* (%)	
Arrest location, *n* (%)		>2	174 (38.16)
PICU	248(54.39)	≤2	282 (61.84)
Inpatient ward	139(30.48)	Sodium bicarbonate, *n* (%)	183 (40.13)
CICU	43(9.43)	Calcium, *n* (%)	52 (11.40)
Other	26	Atropine, *n* (%)	33 (7.24)
Time from hospital admission to event, *n* (%)		Creatine phosphate sodium, *n* (%)	86 (18.86)
<1 h	17 (3.73)	Vitamin C, *n* (%)	38 (8.33)
1 to <6 h	43 (9.43)	Defibrillation, *n* (%)	33 (7.24)
6 to <24 h	66 (14.47)	Rearrest, *n* (%)	136 (29.82)
24 h to <1 wk	150 (32.89)	ECMO, *n* (%)	0 (0)
1 wk or more	180 (39.47)		

^1^ is defined as 7 pm Friday to 6:59 am Monday; ^2^ is defined as 7 pm to 6:59 am.

**Table 2:** Logistic regression analysis.

Variables	Univariate ^1^		Multivariable ^1^	
	*p*	OR (95%CI)	*p*	OR (95%CI)
Age category (vs. 28 d–1 y)				
1–8 y	<0.001	3.06 (1.93~4.84)	0.375	1.39 (0.67~2.89)
≥8 y	<0.001	3.58 (1.89~6.78)	0.087	2.26 (0.89~5.76)
Illness Category (vs. General medical)				
General surgical	0.181	2.44 (0.66~8.99)	0.650	0.65 (0.10~4.23)
Surgical cardiac	0.002	0.29 (0.13~0.64)	<0.001	0.06 (0.02~0.24)
Medical cardiac	0.553	1.37 (0.49~3.81)	0.739	0.75 (0.14~3.98)
Trauma	0.619	1.21 (0.57~2.57)	0.702	0.80 (0.24~2.65)
Respiratory support in place before arrest (vs. None)				
Invasive mechanical ventilation	0.004	1.84 (1.21~2.79)	0.380	2.62 (0.30~22.61)
Noninvasive ventilation	0.018	0.16 (0.04~0.74)	0.519	0.55 (0.09~3.41)
Vasoactive infusion in place before arrest	<0.001	2.79 (1.85~4.20)	<0.001	7.69 (3.49~16.93)
Night	0.003	1.85 (1.23~2.78)	0.565	1.12 (0.65~2.22)
Arrest location (vs. PICU)				
Inpatient ward	0.004	0.51 (0.32~0.81)	0.820	0.88 (0.30~2.59)
CICU	<0.001	0.15 (0.07~0.34)	0.329	0.53 (0.15~1.88)
Other	0.054	0.42 (0.17~1.02)	0.772	0.81 (0.19~3.41)
First documented pulseless rhythm (vs. Bradycardia with poor perfusion)				
Asystole/PEA	0.012	2.82 (1.25~6.36)	0.194	2.16 (0.68~6.88)
VF/pVT	0.223	0.52 (0.18~1.49)	0.010	0.15 (0.03~0.63)
Other or Unknown	0.055	0.44 (0.19~1.02)	0.100	0.33 (0.09~1.23)
Category of CPR duration, min (vs. 1–15)				
16–35	<0.001	4.29 (2.36~7.80)	0.848	1.11 (0.39~3.17)
>35	<0.001	13.29 (5.54~31.88)	<0.001	13.92 (3.24~59.82)
Number of epinephrine doses > 2	<0.001	5.98 (3.75~9.54)	0.026	3.12 (1.15~8.46)
Sodium bicarbonate	<0.001	6.20 (3.90~9.86)	0.132	1.88 (0.83~4.28)
Creatine phosphate sodium	0.001	2.47 (1.43~4.27)	0.232	0.57 (0.23~1.43)
Atropine	0.057	2.25 (0.98~5.18)	0.369	0.52 (0.12~2.18)
Vitamin C	<0.001	5.60 (2.13~14.70)	0.720	1.27 (0.34~4.68)
Airway management (vs. Bag-Mask Ventilation)				
ETI in place before arrest	<0.001	9.82 (4.42~21.84)	0.932	1.11 (0.11~11.01)
Emergency intubation at arrest	<0.001	8.07 (3.47~18.75	0.008	5.17 (1.54~17.36)
Rearrest	<0.001	2.70 (1.71~4.26)	<0.001	8.48 (4.11~17.52)
Factors not in Multivariable logistic regression analysis				
Male	0.901	0.98 (0.65~1.45)		
Local	0.198	1.30 (0.87~1.94)		
Prehospital arrest	0.484	1.26 (0.66~2.39)		
Weekend	0.844	1.04 (0.68~1.60)		
Time from hospital admission to event (vs. <1 h)				
1 to <6 h	0.135	0.33 (0.08~1.41)		
6 to <24 h	0.477	0.60 (0.15~2.46)		
24 h to <1 wk	0.113	0.34 (0.09~1.29)		
1 wk or more	0.137	0.37 (0.10~1.38)		
Defibrillation	0.804	1.10 (0.51~2.37)		
Calcium	0.130	1.64 (0.87~3.09)		

^1^ OR and 95% CI were based on the logistic regression model (*n* = 397). The 59 patients who were discharged against medical advice were excluded from the model because of the indeterminacy of their survival outcomes.

## Data Availability

The data that support the findings of this study are available upon reasonable request from the corresponding author. The data are not publicly available due to ethical reasons.

## Abbreviations

The following abbreviations are used in this manuscript:

CA	Cardiac arrest
IHCA	In-hospital cardiac arrest
OHCA	Out-of-hospital cardiac arrest
ROSC	Return of spontaneous circulation
CPR	Cardiopulmonary resuscitation
ECPR	Extracorporeal cardiopulmonary resuscitation
WLST	Withdrawal of life sustaining therapy
DNR	Do not resuscitate
ECMO	Extracorporeal membrane oxygenation
AHA	American Heart Association
GWTG	Get With The Guidelines
PCPC	Pediatric cerebral performance category
PICU	Pediatric intensive care unit
CICU	Cardiac surgery intensive care unit
BMV	Bag-mask ventilation
ETI	Endotracheal intubation
PEA	Pulseless electrical activity
VF	Ventricular fibrillation
pVT	Pulseless ventricular tachycardia
Neuro	Neurological
OR	Odds ratio
CI	Confidence interval
BLS	Basic Life Support
PALS	Pediatric Advanced Life Support
